# Dissolved organic carbon from the upper Rio Negro protects zebrafish (*Danio rerio*) against ionoregulatory disturbances caused by low pH exposure

**DOI:** 10.1038/srep20377

**Published:** 2016-02-08

**Authors:** Rafael M. Duarte, D. Scott Smith, Adalberto L. Val, Chris M. Wood

**Affiliations:** 1 Laboratory of Ecophysiology and Molecular Evolution, National Institute for Amazonian Research, Manaus, AM, Brazil; 2 Department of Chemistry and Biochemistry, Wilfrid Laurier University, Waterloo, ON, N2L 3C5, Canada; 3 Department of Biology, McMaster University, Hamilton, ON L8S 4K1, Canada; 4 Department of Zoology, University of British Columbia, Vancouver, BC V6T 1Z4, Canada; 5 Biosciences Institute, São Paulo State University - UNESP, Coastal Campus, São Vicente, SP, Brazil

## Abstract

The so-called “blackwaters” of the Amazonian Rio Negro are rich in highly coloured dissolved organic carbon (DOC), but ion-poor and very acidic, conditions that would cause fatal ionoregulatory failure in most fish. However these blackwaters support 8% of the world’s ichthyofauna. We tested the hypothesis that native DOC provides protection against ionoregulatory dysfunction in this extreme environment. DOCs were isolated by reverse-osmosis from two Rio Negro sites. Physico-chemical characterization clearly indicated a terrigenous origin, with a high proportion of hydroxyl and phenolic sites, high chemical reactivity to protons, and unusual proteinaceous fluorescence. When tested using zebrafish (a model organism), Rio Negro DOC provided almost perfect protection against ionoregulatory disturbances associated with acute exposure to pH 4.0 in ion-poor water. DOC reduced diffusive losses of Na^+^ and Cl^−^, and promoted a remarkable stimulation of Na^+^ uptake that otherwise would have been completely inhibited. Additionally, prior acclimation to DOC at neutral pH reduced rates of branchial Na^+^ turnover, and provided similar protection against acid-induced ionoregulatory disturbances, even if the DOC was no longer present. These results reinforce the important roles that DOC molecules can play in the regulation of gill functions in freshwater fish, particularly in ion-poor, acidic blackwaters.

The dissolved component (DOM, dissolved organic matter) of aquatic natural organic matter (NOM) is now recognized to regulate many abiotic and biotic processes in freshwater systems^1^. Functionally, DOM is separated by 0.45-μm filtration, and quantified as dissolved organic carbon (DOC)^2^. For simplicity, we refer to DOM as DOC, recognizing that it contains approximately 50% carbon by mass. Important DOC functions include controlling transport, distribution and accumulation of ions and metals in various environmental compartments^3^,^4^, as well as promoting both indirect and direct physiological impacts on aquatic organisms^5^. DOC is derived from the decomposition of lignin-rich plant material and dead organic biomass, and also synthesis by aquatic microorganisms^2^. DOC molecules have a generally irregular chemical structure and wide range of molecular weights (MW)^6^. The major components of aquatic DOC are “humic substances”, usually representing 50–90% of total content. These are a heterogeneous combination of higher MW “humic” acids and lower MW “fulvic” acids. Humic substances contain a variety of carboxylic, phenolic and carbonyl groups that are associated with the functional properties of DOC molecules in aquatic systems^2^,^7^,^8^. Other lower abundance components, such as amino acids (e.g. tyrosine, tryptophan) may also be important^2^.

All DOCs are not alike. In general, allochthonous (terrigenous) DOCs, derived from the degradation of land-based plant materials, are darker and higher in MW than the autochthonous DOCs synthesized in water bodies by endogenous aquatic microorganisms. Many functional properties of DOCs, such as their affinity for protons and metal ions^9^, surface activity effects^1^, and ability to bind to biological membranes^10^, may be related to optical and physico-chemical characteristics^11^,^12^,^13^,^14^. In turn, functional consequences for aquatic organisms, such as the ability of a particular DOC to protect against metal toxicity^7^,^15^,^16^,^17^,^18^,^19^,^20^,^21^, and to exert effects on ionoregulatory physiology^22^,^23^,^24^,^25^ may be related to these same characteristics. In general, the darker and larger the DOM molecules, the greater are both protective effects against metal toxicity and physiological effects on ionoregulation^5^. Recently, Al-Reasi *et al.* (2013)^8^ related these two functions to the chemical reactivity of DOC to protons, as captured by a Proton Binding Index (PBI), which in turn was strongly correlated to colour originating from aromatic groups.

The blackwaters of the Rio Negro, the major tributary to the Amazon, contain some of the most darkly coloured and abundant DOCs in the world, typically 8-12 mg C L^−1^, but up to 35 mg C L^−1^ in small streams^2^,^26^. These waters are also highly acidic (pHs 3.0-5.5) and so low in essential ions (Na^+^, Cl^−^, Ca^2+^ < 50 μmol L^−1^) that Sioli (1984)^27^ characterized them as ‘slightly contaminated distilled water’. Most fish, if exposed to these pHs and ion concentrations, would quickly die from ionoregulatory failure, due to inhibition of active ion uptake and acceleration of passive ion losses at the gills^28^,^29^. Yet approximately 8% of the world’s fish species are endemic to these blackwaters^30^. This has led to the hypothesis that Rio Negro DOCs have unique protective properties that allow fish to avoid ionoregulatory dysfunction in this extreme environment^31^,^32^,^33^.

To date, support for this hypothesis has been only circumstantial. Several studies have shown that native fish, when exposed to low pH in native Rio Negro water, exhibit better ionoregulatory performance than when exposed in synthetic waters of similar ionic composition but lacking DOC^31^,^32^,^34^. However, when a commercial DOC was tested, it actually exacerbated ionoregulatory failure in one study^34^ yet protected in another^33^. Therefore, our goal was to test the hypothesis by isolating Rio Negro DOC by reverse-osmosis, characterizing its optical and physico-chemical properties by a range of techniques^8^,^16^, and then evaluating whether it protected against ionoregulatory dysfunction in fish exposed to low pH in typical ion-poor water. We used the zebrafish, a non-native model organism^35^, because the disturbances of its ionoregulatory physiology during low pH exposure in the absence of DOC have been studied extensively^29^,^36^,^37^,^38^. Our results confirm the hypothesis, showing remarkable protective actions of upper Rio Negro DOC against ionoregulatory dysfunction in zebrafish at low pH in ion-poor water, effects which may be explained by the unusual physico-chemical characteristics of this DOC.

## Results and Discussion

The physico-chemical properties of Rio Negro DOC isolates were determined in samples from two different sources, representing the aquatic systems of the upper and lower Rio Negro respectively, São Gabriel da Cachoeira (SGC) and Novo Airão (NA) The properties of the two isolates were similar, but those of the DOC from the SGC source were particularly extreme and unusual relative to other sources that our group has characterized by the same techniques^8^,^16^ (Table 1). The very high specific absorbances (SAC_340_) indicate the presence of a high content of ringed chromophores^11^,^15^ in both Rio Negro DOC samples, in accordance with their dark colour. Indeed the value of SAC_340_ for SGC was almost twice that of a DOC from another well-characterized, highly terrigenous source, a peat bog in Canada (Luther Marsh). Furthermore, the fluorescence indices (FI) were quite low, indicative of terrestrial origin^12^, and Abs_254/365_ values were very low, signalling a high mean MW for the DOC molecules^13^. Overall, these indices suggest large lignin-degradation products with high levels of aromatic humic and fulvic acids enriched in carboxylic and phenolic groups. Parallel factor analysis (PARAFAC) reinforced these conclusions, with humic-like components predominating in the excitation-emission matrices (EEM), followed by fulvic-like moieties, though the humic component did not dominate to the same extent as in some other terrigenous isolates such as Luther Marsh or Nordic Reservoir, and the tryptophan- and tyrosine-like components were not insignificant, suggesting some autochthonous input. One possible origin for these protein-like signals is violacein, a purple pigment produced by *Chromobacterium violaceum*, a microbe which is abundant in Rio Negro waters; additionally, this would contribute to the SAC_340_ signal^39^.

The acid-base properties revealed by titration of Rio Negro DOC samples, specifically their acidity constants (pK_a_) and their densities (L_T_, mmol mg^−1^), were similar to those previously reported by Al-Reasi *et al.* (2013)^8^ for DOC molecules from other terrigenous sources such as such as Nordic Reservoir and Luther Marsh (Table 1). However, the Rio Negro DOCs exhibited lower acidic peaks and higher basic peaks, which suggests a lesser contribution of carboxylic sites. Thus, hydroxyl and phenolic sites likely occur in higher proportion in these Rio Negro DOCs, than in either Nordic Reservoir or Luther Marsh DOC. The presence of two intermediate peaks in the pK_a_ spectra was another important feature of Rio Negro DOC (Supplementary Fig. S1 online). These likely represent the contribution of proteinaceous material, such as tryptophan-like and tyrosine-like fluorophores that are not usually seen in DOC molecules from other terrigenous sites. The values for the Proton Binding Index (PBI) were high for both Rio Negro isolates, especially SGC DOC (Table 1), which fits with the prediction that the darker the organic matter, the greater is their PBI^8^. Both the spectroscopic features of DOC^5^,^24^ and the PBI^8^ have proven to be useful (and correlated) predictors of the ability of DOC molecules to interact directly with the gills of fish^5^. Interestingly, these same properties also correlate with protective effects against metal toxicity in freshwater organisms^7^,^8^,^16^,^17^,^19^,^20^,^21^ (see Supplementary Fig. S2 online for additional information). Based on the more pronounced physico-chemical properties, we selected SGC DOC for all experimental tests with zebrafish.

**Figure 1:**
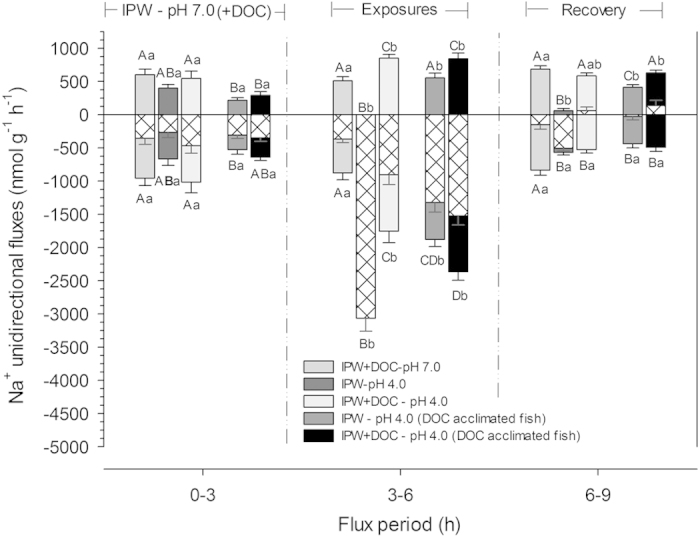
Unidirectional sodium influx (J^Na^_in_, upward positive solid bar), unidirectional sodium efflux (J^Na^_out_, downward negative solid bars) and net sodium flux rates (J^Na^_net_, cross-hatched bars) of adult zebrafish in ion-poor water (IPW). Means ± 1 SEM (N = 10 in each treatment). In the left-hand panel, the first three sets of bars represent fish initially tested (0-3 h) under the same control condition (no DOC) to which they were all acclimated (IPW – pH 7.0), and then in the middle panel acutely exposed (3–6 h) to either IPW + DOC – pH 7.0, or IPW – pH 4.0, or IPW + DOC – pH 4.0, followed in the right-hand panel by a recovery period (6–9 h) in which all fish were again exposed to the common acclimation condition (IPW – pH7.0). In addition, the fourth and fifth bars represent sodium flux rates of zebrafish which had been acclimated to DOC at pH 7.0 for two weeks prior to test. In the left-hand panel, these fish were initially tested (0–3 h) under their common acclimation condition (IPW + DOC – pH 7.0), and then in the middle panel acutely exposed (3–6 h) to either IPW – pH 4.0, or IPW + DOC – pH 4.0, followed in the right-hand panel by a recovery period (6–9 h) in which both groups were again exposed to their common acclimation condition (IPW + DOC – pH 7.0). Upper case letters represent significant differences (p < 0.05) in J^Na^_in_ or J^Na^_out_ among fish under different exposure regimes (different shading schemes) within the same flux period. Lower case letters represent significant differences (p < 0.05) in J^Na^_in_ or J^Na^_out_ of animals in the same regime of exposure (bars with same shading scheme), among different flux periods. Bars sharing the same letter are not significantly different.

**Figure 2:**
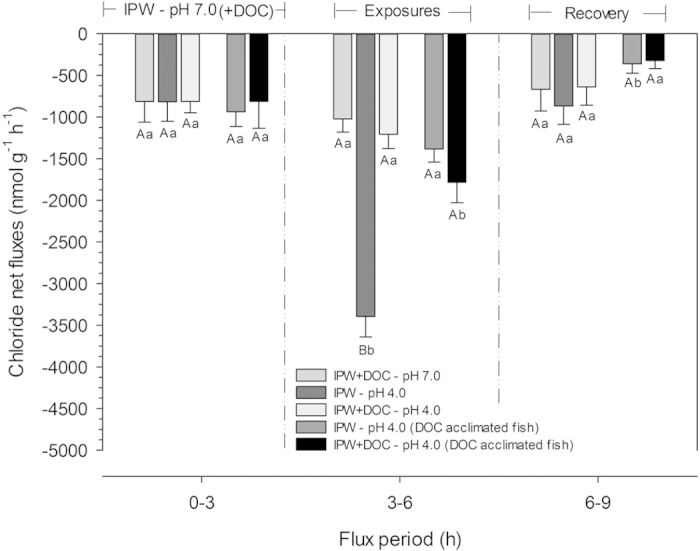
Net chloride flux rates (J^Cl^_net_) of adult zebrafish in ion-poor water (IPW). Means ± 1 SEM (N = 10 in each treatment). In the left-hand panel, the first three bars represent fish initially tested (0-3 h) under the same control condition (no DOC) to which they were all acclimated (IPW – pH 7.0), and then in the middle panel acutely exposed (3–6 h) to either IPW + DOC – pH 7.0, or IPW – pH 4.0, or IPW + DOC – pH 4.0, followed in the right-hand panel by a recovery period (6–9 h) in which all fish were again exposed to the common acclimation condition (IPW – pH7.0). In addition, the fourth and fifth bars represent J^Cl^_net_ values of zebrafish which had been acclimated to DOC at pH 7.0 for two weeks prior to test. In the left-hand panel, these fish were initially tested (0–3 h) under their common acclimation condition (IPW + DOC – pH 7.0), and then in the middle panel acutely exposed (3–6 h) to either IPW – pH 4.0, or IPW + DOC – pH 4.0, followed in the right-hand panel by a recovery period (6–9 h) in which both groups were again exposed to their common acclimation condition (IPW + DOC – pH 7.0). Statistical significance (p < 0.05) is shown as in Fig. 1.

Series 1 evaluated the potential protective effects of Rio Negro DOC on the ionic balance of zebrafish acutely exposed to low pH. Exposure to pH 4.0 in ion-poor water without DOC (IPW – pH 4.0) caused a complete blockade of unidirectional sodium influx (J^Na^_in_), as well as a very large stimulation of unidirectional sodium efflux (J^Na^_out_), when compared with rates of fish at ion-poor water at pH 7.0 (IPW – pH 7.0) (Fig. 1). Therefore net sodium flux (J^Na^_net_) became highly negative, equal to J^Na^_out_. Upon return to pH 7.0, J^Na^_out_ recovered completely, but J^Na^_in_ remained substantially depressed for at least 3 h (Fig. 1). Net fluxes of Cl^−^ (J^Cl^_net_) became similarly negative during exposure to IPW- pH 4.0, increasing more than 3-fold, but recovered fully upon return to pH 7.0 (Fig. 2). These results agree with many previous studies that have also reported reduced influxes, and increased diffusive effluxes and net losses of Na^+^ and Cl^−^ in both temperate and tropical fish exposed to low pH, especially in ion-poor water^28^,^29^,^33^,^37^,^38^,^40^,^41^,^42^,^43^. However, the presence of SGC DOC (10 mg L^−1^) greatly ameliorated these effects, which helps to explain how many fish species can thrive in acidic, ion-poor Rio Negro water. The increase in J^Na^_out_ during pH 4.0 exposure was reduced by 50% in the presence of SGC DOC (IPW + DOC – pH 4.0), and the blockade of J^Na^_in_ was replaced by a stimulation, such that there was no longer a significant change in J^Na^_net_ (Fig. 1). SGC DOC also completely eliminated the elevation in the negative J^Cl^_net_ (Fig. 2).

Previous studies have shown qualitatively similar but smaller protective effects when comparing the responses of fish in Rio Negro water with responses in synthetic ion-poor water of similar ionic composition but lacking DOC^31^,^32^,^43^. These authors speculated that DOC was involved, but Wood *et al.* (2003)^43^ reported that experimental addition of a commercially available DOC (Aldrich humic acid) actually exacerbated the effects of low pH exposure. Following up this previous finding, the present study is the first to demonstrate that it is the specific DOC native to the Rio Negro (or some component thereof) that is the protective agent against disturbances in both the active influx and diffusive efflux components, effects which are seen even in a non-native fish species.

How might this work? Traditionally, diffusive ion losses at low pH were thought to occur mainly through the paracellular pathways in the gills due to a leaching of Ca^2+^ and accompanying disturbance of transmembrane tight junction proteins such as occludins and claudins^28^,^38^. Recently, increased paracellular permeability and the protective role of water Ca^2+^ were directly confirmed in zebrafish exposed to low pH^29^,^37^. In Rio Negro fish in synthetic water at low pH, experimental increases in water Ca^2+^ concentration have protective effects^31^,^32^,^43^ similar to those seen with DOC in the present study. This suggests that in Ca^2+^ -poor waters, Rio Negro DOC molecules can rapidly modulate the tightness of the gill epithelium of zebrafish, perhaps through Ca^2+^ -like effects on tight junction integrity and/or through post-translational regulation of claudins and occludins^44^. A seminal study by Campbell *et al.* (1998)^10^ demonstrated that DOC molecules can actually bind to the surface membranes of isolated gill cells; this phenomenon was favoured by low pH, suggesting a hydrophobic bonding or a hydrogen-bonding sorption mechanism. Presumably, the negatively charged sites on DOC are titrated by the increased H^+^ concentration, making it easier for these amphiphilic DOC molecules to bind to key sites such as tight junctions. The high proportion of hydroxyl and phenolic sites, high chemical reactivity to protons (indicated by PBI), and unusual proteinaceous content of SGC DOC may all contribute to this property. DOC-binding may stabilize the junctions in the same way as Ca^2+^ ions (now displaced by low pH), thereby reducing passive paracellular Na^+^ and Cl^−^ effluxes, or even altering the transcellular permeability (see below).

Protection by SGC DOC against the inhibitory effects of pH 4.0 on active Na^+^ influx (J^Na^_in_) was even more impressive (Fig. 1). In tests with “laboratory waters”, J^Na^_in_ is inhibited by acute exposure to this level of acidity in almost all freshwater teleosts, except in some native to Rio Negro blackwaters^28^,^29^,^33^. Clearly, the immediate complete protection, indeed overcompensation, against this effect in zebrafish by the presence of realistic levels of SGC DOC (Fig. 1), suggests some type of fast physicochemical interaction. Possibilities include higher pH or higher Na^+^ levels in the gill boundary layer due to the presence of bound DOC molecules, hindered access of external H^+^ ions to Na^+^ gill transport sites, favourable changes in transepithelial potential^24^, and/or direct solubilisation of DOC molecules into branchial lipoprotein cell membranes^45^, thereby changing fluidity and transporter characteristics in the transcellular pathway. However the prior acclimation experiments of Series 2 (see below) suggest that these acute physicochemical interactions affecting permeability, uptake, or both may not be the whole story.

Series 2 tested whether prior acclimation of zebrafish to Rio Negro DOC would confer protection against disturbances of ionic fluxes caused by low pH. Acclimation to SGC DOC resulted in significantly lower J^Na^_in_ and J^Na^_out_ values at pH 7.0 (i.e. lower Na^+^ turnover), with no change in J^Na^_net_ (Fig. 1) or J^Cl^_net_ (Fig. 2), in comparison to fish acclimated to IPW–pH 7.0 with no DOC. Acute exposure to both IPW – pH 4.0 and IPW + DOC–pH 4.0 resulted in similar responses, revealing protective effects of prior DOC exposure against acidity, which were present regardless of the presence or absence of DOC during the low pH exposure. These included significant increases in J^Na^_in_, lesser elevations in J^Na^_out_, substantially attenuated net losses of Na^+^ and Cl^−^, and more complete recovery, similar to the protective effects seen when DOC was presented only acutely (Figs 1 and 2).

**Figure 3:**
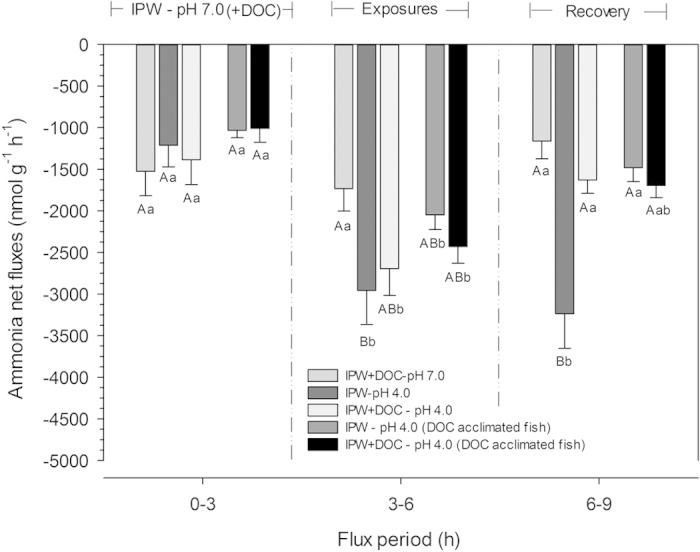
Net ammonia flux rates (J^Amm^_net_) of adult zebrafish in ion-poor water (IPW). Means ± 1 SEM (N = 10 in each treatment). In the left-hand panel, the first three bars represent fish initially tested (0–3 h) under the same control condition (no DOC) to which they were all acclimated (IPW – pH 7.0), and then in the middle panel acutely exposed (3–6 h) to either: IPW + DOC – pH 7.0, or IPW – pH 4.0, or IPW + DOC – pH 4.0, followed in the right-hand panel by a recovery period (6–9 h) in which all fish were again exposed to the common acclimation condition (IPW – pH 7.0). In addition, the fourth and fifth bars represent J^Amm^_net_ values of zebrafish which had been acclimated to DOC at pH 7.0 for two weeks prior to test. In the left-hand panel, these fish were initially tested (0–3 h) under their common acclimation condition (IPW + DOC – pH 7.0), and then in the middle panel acutely exposed (3–6 h) to either IPW – pH 4.0, or IPW + DOC – pH 4.0, followed in the right-hand panel by a recovery period (6–9 h) in which both groups were again exposed to their common acclimation condition (IPW + DOC – pH 7.0). Statistical significance (p < 0.05) is shown as in Fig. 1.

These results suggest direct effects of long-term acclimation to SGC DOC on both permeability and transport processes, effects which can persist even when the DOC is no longer present in the water. To our knowledge, such phenomena have not been reported previously. These could occur because DOC molecules remain persistently bound to gill sites for some time, involving any or all of the acute protective mechanisms suggested above, or because their presence has elicited persistent physiological or structural changes in the gills. With respect to the latter, increased branchial Na^+^, K^+^ -ATPase activity^46^ and altered Na^+^, Cl^−^ and Ca^2+^ uptake kinetics^22^,^23^,^25^,^43^ have been seen in organisms exposed to DOC for various periods. Recent evidence indicates that the stress hormone cortisol plays a critical role in allowing zebrafish to acclimate to low pH over the longer term^47^. Is it possible that the phenolic ring structure of SGC DOC mimics the action of cortisol? In current models of gill transport functions in freshwater fish, Na^+^uptake is linked to ammonia excretion via a metabolon involving Rhesus (Rh) glycoproteins, Na^+^ and H^+^ transporters, and carbonic anhydrase^48^,^49^,^50^. Especially in ion-poor, acidic waters, ammonia excretion plays a key role in driving Na^+^ uptake^29^,^36^,^37^,^51^. Yet in the absence of DOC, the complete blockade of J^Na^_in_ by exposure to IPW-pH 4.0 in Series 1 (Fig. 1) was paradoxically accompanied by a substantial increase in net ammonia excretion (J^Amm^_net_). This apparent uncoupling upon acute exposure to low pH has been reported frequently, and explained by H ^+^ blockade of Na^+^ uptake and simultaneous increased passive diffusion of NH_3_, facilitated by acid-trapping in the boundary layer of the gill epithelium (reviewed by Wilkie, 2002)^52^. Damage may also be involved, because both the inhibition of J^Na^_in_ and the stimulation of J^Amm^_net_ were also seen during the recovery period (i.e. IPW – pH 7.0; Figs 1 and 3). Yet the presence of SGC DOC during the acid-exposure period actually stimulated J^Na^_in_ while allowing the increase in J^Amm^_net_ to still occur, and facilitated the rapid recovery process for both flux rates. Prior acclimation to DOC in Series 2 resulted in very similar responses during the acid exposure and recovery periods (Figs 1 and 3).

**Figure 4:**
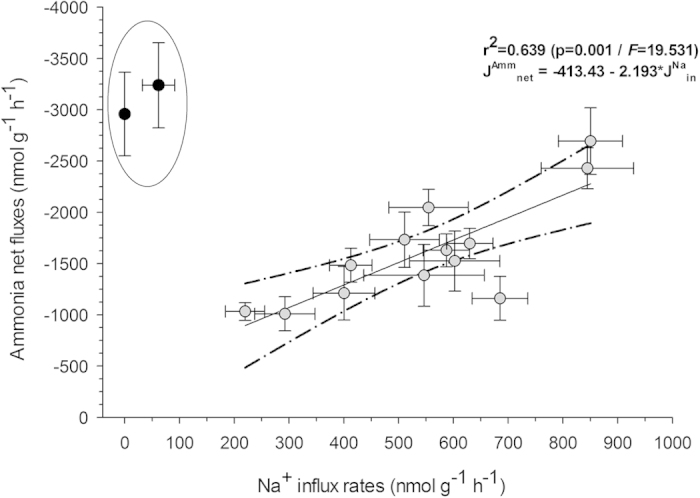
The relationship between net ammonia flux rates (J^Amm^_net_) and unidirectional Na^+^ uptake rates (J^Na^_in_) of adult zebrafish in different exposure conditions in ion-poor water (IPW). Means ± 1 SEM. Gray circles represents flux rates of both J^Amm^_net_ and J^Na^_in_ of zebrafish under the acclimation conditions (i.e. IPW – pH 7.0 or IPW + DOC – pH7.0; 0–3 h), and then acutely exposed to either IPW + DOC – pH 7.0, or IPW – pH 4.0 or IPW + DOC – pH 4.0 (3–6 h), followed by a recovery period in which they were again exposed to their acclimation condition (IPW – pH 7.0 or IPW + DOC – pH 7.0). Note that the two black circles (not used in the regression) represent data from fish acutely exposed to IPW – pH 4.0 (no DOC), and these same fish during the recovery period at IPW – pH 7.0, where J^Amm^_net_ was entirely uncoupled from J^Na^_in_. Nonlinear regression analysis was performed using *Sigma Plot* v 11.0. r^2^ = 0.639; *p* = 0.001, *F* = 19.531.

DOC appears to maintain the coupling of Na^+^ uptake to ammonia excretion in zebrafish during and after exposure to low pH in ion-poor water. Indeed, J^Amm^_net_ was correlated with J^Na^_in_ in gills of zebrafish under all experimental conditions (r^2^ = 0.639; Fig. 4), except under IPW – pH 4.0, in fish not acclimated to DOC, where the processes were uncoupled during and after acid exposure. The upregulation of J^Amm^_net_ (Fig. 3) has been reported as a compensatory response to enhance J^Na^_in_ in zebrafish in the face of elevated diffusive Na^+^ losses during acid exposure^29^,^36^,^37^. However, in these studies, the phenomena developed slowly (hours to days) and the possible involvement of DOC was not investigated. The present results suggest that DOC supports functional coupling of Na^+^ uptake to ammonia excretion via the Rh metabolon^48^,^53^ allowing an immediate compensatory response, but the mechanism awaits future investigation.

In conclusion, this study provides clear evidence confirming the hypothesis^31^,^32^,^33^ that Rio Negro DOC protects freshwater fish against ionoregulatory disturbances associated with acute low pH exposure in ion-poor water. This protection includes both controlling the “tightness” of the gills so as to reduce diffusive losses of Na^+^ and Cl^−^ during acid stress, and promoting a remarkable stimulation of Na^+^ uptake that otherwise would have been completely inhibited. The latter seems to involve maintenance of a functional coupling whereby increased ammonia excretion can drive elevated Na^+^ uptake during low pH exposure. Interestingly, prior acclimation to SGC DOC at neutral pH reduces rates of branchial Na^+^ turnover, and provides similar protection against acid-induced ionoregulatory disturbances. The latter occurs even if the DOC is no longer present, suggesting that acclimation to DOC induces persistent changes in gill physiology which provide greater tolerance to low pH. These results reinforce the important roles that DOC molecules can play in the regulation of gill functions in freshwater fish, and their critical importance for life in ion-poor, acidic blackwaters. In future, it will be of interest to evaluate if the pattern of ionoregulatory responses seen in zebrafish to DOC is widespread in other teleost fish species, particularly in those fish inhabiting acidic ion-poor waters, and also its correlation to specific structural properties of DOC from distinct aquatic environments.

## Methods and Materials

### Experimental animals and holding

Adult zebrafish (0.377 ± 0.10 g) were purchased from Pets Mart (Hamilton, Canada), fed daily to satiation with a commercial food (Newlife Spectrum, Homestead, USA), maintained on a 12 h/12 h light/dark regime and kept in 50-liter aquaria for one month in moderately hard Lake Ontario water (Na^+^ 600 μM, Cl^−^ 800 μM, K^+^ 50 μM, Ca^2+^ 900 μM and Mg^2+^300 μM). After this first acclimation period, 50% of the water was replaced daily with reconstituted ion-poor water (IPW) until the desired final composition was reached (Na^+^ 50 μM, Cl^−^ 80 μM, K^+^ 15 μM, Ca^2+^ 10 μM and Mg^2+^3 μM) simulating the ion-poor levels of natural Rio Negro water^30^. Fish were allowed to acclimate for at least 1 week to this IPW condition.

All the experimental procedures and protocols using zebrafish were previously approved by the McMaster University Animal Research Ethics Board (AUP 12-12-45), and were performed in accordance with the guidelines on “The care and use of fish in research, teaching and testing” of the Canadian Council for Animal Care (2005).

### Collection and characterization of Rio Negro DOC

DOCs were from two pristine sites representing the upper and lower Rio Negro: São Gabriel da Cachoeira (SGC) district and Novo Airão (NA) city, 850 km and 180 km upstream from Manaus, respectively (see Supplementary Table S1 for water chemistry). At each site, water from the main channel of the Rio Negro was pumped through 1-μm wound string filters to a reverse-osmosis unit (Vontron^®^ULP21-4021 polyamide membrane, Permution, model PEOS-0001, Curitiba, Brazil)^54^,^55^. After collection, the NOM concentrates were treated with a cation exchange resin (Amberlite IR-118 (H), Sigma-Aldrich, St. Louis, USA), to avoid interferences by cations built up during reverse-osmosis^7^. Concentrates were then 0.45-μm filtered (Acrodisc^TM^, Pall, Ann Arbour, USA), stored at 4 °C, and characterized for physico-chemical properties and/or used in live fish experiments.

Acid-base titrations employed DOC isolates (68.36 ± 1.96 mg C L^−1^) diluted in 0.01 M KNO_3_ (Sigma-Aldrich, St. Louis, MO, USA). Base (0.1 N NaOH, from a standardized 1.005 N NaOH stock, Sigma-Aldrich) was added to stirred DOC solutions (pH 3.0) so as to increase pH in ~0.1-unit intervals until pH 11.0. Five titration replicates were carried out for each DOC sample, as well as three titration replicates with Epure^TM^ water (MilliQ, Millipore, Etobicoke, Canada) acidified with 1.000 N hydrochloric acid (HCl, Sigma-Aldrich) to standardize the NaOH titrant. Proton binding constants (pK_a_) and their site densities (L_T_, μmol mg^−1^) were determined through a fully optimized continuous model (FOCUS) using in-house Matlab^TM^ (Mathworks, Natick, USA) programs^56^. Binding site densities within a specific pK_a_ range were determined by integration of the area under the curve in the pK_a_ spectrum.

For optical measurements, isolates were diluted with Epure^TM^ water to 10 mg C L^−1^ and pH adjusted to ~7.0 (0.1 N NaOH). The specific absorbance coefficient at 340 nm (SAC_340_) was determined as an indicator of the aromatic composition^11^, while the fluorescence index (FI) was used as an indicator of DOC origin^12^. The ratio of absorbance at 254 nm to that at 365 nm (Abs_254/365_) was measured as an indicator of MW^13^. Full excitation-emission matrices (EEMs) were generated and subjected to parallel factor analysis (PARAFAC) that quantitatively partitions the origin of the fluorescence^14^,^57^. The spectral EEMs were modeled using the PLS Toolbox from Eigenvector Research Inc. (Wenatchee, WA, USA) running on a Matlab^TM^ platform. PARAFAC assigned the fluorescence on a percentage basis based on the *a priori* assumption that there were four components (humic-like, fulvic-like, tyrosine-like, and tryptophan-like)^7^,^8^.

### Experimental design for flux measurements

Following the characterization of DOCs from the two Rio Negro sites, we selected SGC DOC for all experimental tests because of its more distinctive physicochemical properties. DOC concentrate was diluted (to a nominal concentration of 10 mg C L^−1^) with reconstituted ion-poor water, and test solutions were stored in the dark for 24 h^21^. The final pH of all experimental solutions was adjusted to neutral (pH 7.0; 0.01 N KOH) or acid (pH 4.0; 0.01 N HNO_3_) as appropriate. Throughout the experiments, pH values in all chambers were adjusted to the desired level (neutral or acidic) with 0.001 N KOH or 0.001 N HNO_3_ when necessary (see Supplementary Table S2 for pH, DOC, and water ions in experimental solutions).

For experiments, fish (N = 10 per treatment) were transferred from the holding aquaria to individual 40-ml aerated chambers filled with reconstituted ion-poor water representing the control condition (see below for details) for a 1-h settling period. Then 0.01 μCi ml^−1^ of ^22^NaCl (Amersham, Little Chalfont, U.K.) was added to each chamber. Following 5 min of mixing by aeration, a 3-h flux measurement was started with 6-ml samples taken at 0 h and 3 h. After the first 3-h flux period, water in each chamber was removed with a 60-ml syringe, taking care not to air-expose the fish, and replaced with a fresh reconstituted ion-poor water solution representing one of the experimental conditions. Again, 0.01 μCi ml^−1^ of ^22^NaCl was added, and following 5 min of mixing, another 3-h flux measurement was carried out. Following the second 3-h flux period, water in the chambers was changed again, back to fresh ion-poor water at pH 7.0, and after addition of radioisotope, a 3-h recovery flux measurement was performed. Water samples were kept at 4 °C prior to measurements of ^22^Na radioactivity, and total Na^+^, Cl^−^ and ammonia. After the experiments, fish were weighed and monitored; no mortalities occurred under any of the experimental conditions tested.

The goal of Series 1 was to test whether DOC, presented simultaneously with low pH, would protect fish against ionoregulatory disturbances during acute exposure to pH 4.0. Therefore, in the control period, all three groups were exposed to the same water quality, ion-poor water at pH 7.0. In the experimental period, the three treatments were ion-poor water plus DOC at pH 7.0 (IPW + DOC – pH 7.0), ion-poor water with no DOC at pH 4.0 (IPW – pH 4.0), and ion-poor water plus DOC at pH 4.0 (IPW + DOC – pH 4.0) so as to assess the separate and combined effects of acid exposure and DOC exposure. During the recovery period, all three groups were exposed to soft water at pH 7.0 with no DOC.

The goal of Series 2 was to test whether prior acclimation to DOC would protect zebrafish against acute exposure to pH 4.0. To this end, animals were acclimated for 2 weeks to IPW + DOC - pH 7.0 (8 mg C L^−1^ of SGC DOC), prior to the experimental exposures. All experimental procedures were conducted as described above, but in the first 3-h flux period, both groups were exposed to the acclimation condition, IPW + DOC – pH 7.0. The 3-h experimental treatment was either IPW – pH 4.0 (i.e. no DOC) or IPW + DOC – pH 4.0, followed by a final 3-h recovery period for both groups in IPW + DOC – pH 7.0. The two experimental conditions served to differentiate effects dependent on the continued presence of DOC from those acquired entirely from the prior acclimation to DOC.

### Sodium unidirectional fluxes and chloride and ammonia net fluxes

Unidirectional and net Na^+^ flux rates (in nmol g^−1^ h^−1^) were measured according to Wood (1992)^58^. ^22^Na radioactivities in all water samples were determined using a Wizard 1480 Auto Gamma Counter (Perkin Elmer, Waltham, USA), and total Na^+^ concentrations using atomic absorption spectrophotometry (Varian SpectrAA 220FS, Mulgrave, Australia). Briefly, mean specific activity (SA) of the radioisotope (cpm nmol^−1^) in water samples was determined as the mean ratio between the concentration of ^22^Na radioactivity (cpm ml^−1^), and the concentration of total Na^+^ in the water (nmol ml^−1^) during the flux period. Unidirectional influx rates (J^Na^_in_) of fish during each period were calculated as:


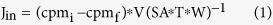


where cpm_i_ = radioisotope cpm ml^−1^ at the beginning of flux period, cpm_f_ = radioisotope cpm ml^−1^ at the end of flux period, V = volume of water in the experimental chamber (ml), T = flux period (h) and W = wet mass of fish (g).

Total Cl^−^ and ammonia concentrations in water samples were determined colorimetrically through the mercury thiocyanate^59^ and salicylate/hypochlorite methods^60^, respectively. The net flux rates (J_net_) of Na^+^, Cl^−^ and ammonia were calculated as:


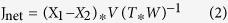


where X_1_ and X_2_ were, respectively, the initial and final Na^+^, Cl^−^ or total ammonia concentrations (nmol ml^−1^) in the water during the flux period. Unidirectional efflux rates (J_out_) were calculated as:


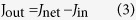


### Statistical analyses

All data are reported as means ± 1 s.e.m. (N = 10). Statistical significance was accepted at p < 0.05. Significant differences in Na^+^ influx (J_in_), efflux (J_out_), and net flux rates (J_net_), and also in both Cl^−^ and ammonia J_net_ values, were determined through a one-way ANOVA, followed by the *a posteriori* Dunnett’s multiple comparison test. In the case of a failed normality test, a non-parametric Kruskal-Wallis test was performed. All statistical analyses and graphics employed Sigma Stat and Sigma Plot software (Jandel Scientific, San Jose, USA).

## Additional Information

**How to cite this article**: Duarte, R. M. *et al.* Dissolved organic carbon from the upper Rio Negro protects zebrafish (*Danio rerio*) against ionoregulatory disturbances caused by low pH exposure. *Sci. Rep.* **6**, 20377; doi: 10.1038/srep20377 (2016).

## Supplementary Material

Supplementary Information

## Figures and Tables

**Table 1:** Summary of physicochemical properties of natural dissolved organic carbon (DOC) samples isolated by reverse osmosis from different freshwater systems.

DOC source	Coordinates	Type	SAC (cm^2^ mg^−1^)^b^	Abs_254/365_^c^	FI^d^	Binding ligand capacities (L_T_, μmol mg^−1^)^e^			
						Acid	Intermediate	Basic	PBI
**Dechlorinated Hamilton tap water (DC)**^a^	—	Tap water isolate	3.72	15.72	1.75	2.56	0.36	2.86	0.13
**Lake Ontario (LO)** a	43°29’N 79°79’W	Autochthonous	4.85	9.75	2.54	1.32	0.50	3.75	0.20
**Bannister Lake (BL)** a	43°30’N 80°83’W	Autochthonous	14.16	6.31	1.51	4.26	0.89	1.79	0.30
**Preston Effluent (PE)** a	43°39’N 80°35’W	Sewage-derived	14.77	5.40	1.94	2.67	0.38	4.08	0.11
**Nordic Reservoir (NR)** a	—	Terrigenous	28.76	4.50	1.21	1.58	0.31	0.79	0.26
**Luther Marsh (LM)** a	43°37’N 80°26’W	Terrigenous	39.30	3.72	1.19	1.74	0.70	1.45	0.44
**Aldrich humic acid (AHA)** a	—	Coal-derived	79.98	2.53	0.83	1.89	0.49	1.17	0.32
**Novo Airão (NA)**	2°37’S 60°56’W	Terrigenous	59.00	2.90	1.42	1.01	0.73	2.89	0.38
**São Gabriel da Cachoeira (SGC)**	0°07’S 67°05’W	Terrigenous	73.00	2.91	1.31	1.21	0.80	1.54	0.58

^a^Data from Al-Reasi *et al.* (2013)^8^.

^b^SAC_340_ is the specific absorbance coefficient at 340 nm normalized to DOC.

^c^Abs_254/365_ is the ratio of absorbance at 254 nm to that at 365 nm.

^d^FI is the fluorescence index.

^e^LT is the binding site densities of DOC molecules. See text for description of each quality parameter.
