# Supplementary material for: Dissolved organic carbon from the upper Rio Negro protects zebrafish (Danio rerio) against ionoregulatory disturbances caused by low pH exposure
Source: Sci Rep. 2016 Feb 8;6:20377. doi: 10.1038/srep20377 (PMC4745052; doi:10.1038/srep20377)
Supplement: Supplementary Information [file srep20377-s1.pdf]

Supplementary Information:

Dissolved organic carbon from the upper Rio Negro protects zebrafish (*Danio rerio*) against ionoregulatory disturbances caused by low pH exposure

*Correspondence to author: rafaelmd@clp.unesp.br

Rafael M. Duarte[1,5*], D. Scott Smith[2], Adalberto L. Val[1], Chris M. Wood[1,3,4]

[1]Laboratory of Ecophysiology and Molecular Evolution, National Institute for Amazonian Research, Manaus, AM, Brazil

[2]Department of Chemistry and Biochemistry, Wilfrid Laurier University, Waterloo, ON, Canada, N2L 3C5

[3]Department of Biology, McMaster University, Hamilton, ON, Canada L8S 4K1

[4]Department of Zoology, University of British Columbia, Vancouver, BC, Canada V6T 1Z4

[5]Biosciences Institute, São Paulo State University - UNESP, Coastal Campus, São Vicente, SP, Brazil

Supplementary Information:

Supplementary Table S1. Physicochemical properties of natural Rio Negro water.

Supplementary Table S2. Mean ionic composition, DOC concentration and pH of all experimental solutions.

Supplementary Figure S1. pKa spectrum results for titrations of SGC and NA DOC samples from the Rio Negro compared to spectra of DOC from other sources.

Supplementary Figure S2. Proton Binding Index (PBI) versus SAC_{340} of both Rio Negro DOC samples and DOC from different sources.

Table S1. Physicochemical properties of natural Rio Negro water (means ± 1 s.e.m.) in both sites, representing the upper and lower Rio Negro environments, where DOC samples were collected.

**Amazon DOC sampling site**	**Temp.**	**O_2**	**Cond.**	**pH**	**DOC**	**Major ion concentrations**				
						**Na^+**	**K^+**	**Ca^{2+}**	**Mg^{2+}**	**Cl^-**
**Novo Airão (Lower Rio Negro)**	30.33 ±0.22	6.59 ±0.07	11.63 ±0.17	4.87 ±0.02	8.69 ±0.12	11.21 ±0.26	9.79 ±0.13	18.05 ±0.10	2.54 ±0.08	19.24 ±0.21
**São Gabriel da Cachoeira (Upper Rio Negro)**	29.50 ±0.57	6.72 ±0.15	13.66 ±0.07	4.41 ±0.01	9.89 ±0.10	14.31 ±0.19	9.54 ±0.26	20.54 ±0.14	2.40 ±0.05	19.17 ±0.12

Temp. = Temperature (°C); O_2 = Oxygen concentration (mg L^{-1}); Cond. = Conductivity (μS cm^{-1}), DOC = Dissolved organic carbon concentration (mg C L^{-1}). All major ion concentrations are expressed in μM. Means ± 1 SEM.

Table S2

Table S2. Mean ionic composition, DOC concentration and pH of all experimental solutions used in flux measurements with zebrafish under two different regimes of acclimation.

**Acclimation regime**	***Exposure conditions***	**pH**	**DOC**	**Na^+**	**Cl^-**	**Ca^{2+}**	**Mg^{2+}**
**Soft-water – pH 7.0 (Series 1)**	*SW – pH 7.0*	6.97±0.3	0.7±0.1	51±1	86±3	10±0.4	3±0.2
	SW+DOC – pH 7.0	7.02±0.4	8.6±0.2	52±1	88±4	11±0.6	3±0.3
	SW – pH 4.0	4.05±0.1	0.6±0.1	50±1	85±4	10±0.3	3±0.1
	SW+DOC – pH 4.0	4.07±0.2	8.7±0.2	51±1	85±3	10±0.4	3±0.2
**Soft-water + DOC – pH 7.0 (Series 2)**	*SW – pH 4.0*	4.07±0.1	0.6±0.1	52±2	83±3	11±0.4	3±0.1
	SW+DOC – pH 4.0	3.98±0.2	8.9±0.2	50±1	87±2	9±0.6	3±0.3

DOC = Concentration of dissolved organic carbon from SGC (upper Rio Negro) in experimental solutions (mg C L^{-1}). All major ions concentrations are expressed in μM. Means ± 1 SEM.

Figure S1.

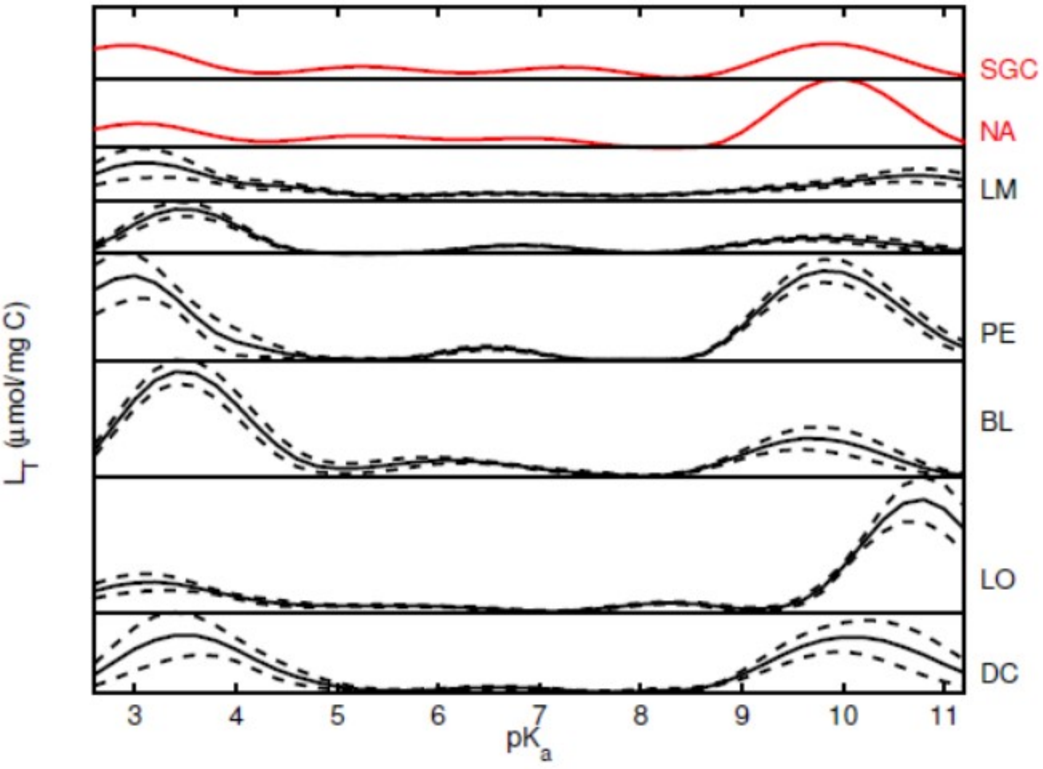

Figure S1. pKa spectrum results for titrations of SGC and NA DOC samples from the Rio Negro compared to spectra of DOC from other sources (data from Al-Reasi et al. 2013)[8]. Overall Rio Negro DOC exhibited similar trends in spectra, with an initial acidic peak for carboxylic acids and a terminal basic peak for phenolic substances. Note that DOC samples from Rio Negro show two intermediate small peaks that are unusual relative to other DOC sources. These likely represent the contribution of proteinaceous material, such as tryptophan-like and tyrosine-like fluorophores that are not usually seen in DOC molecules from other terrigenous sites. DC= Dechlorinated Hamilton tap water; LO= Lake Ontario; BL= Bannister Lake; PE= Preston Effluent; LM= Luther Marsh; NA= Novo Airão; SGC= São Gabriel da Cachoeira.

Figure S2.

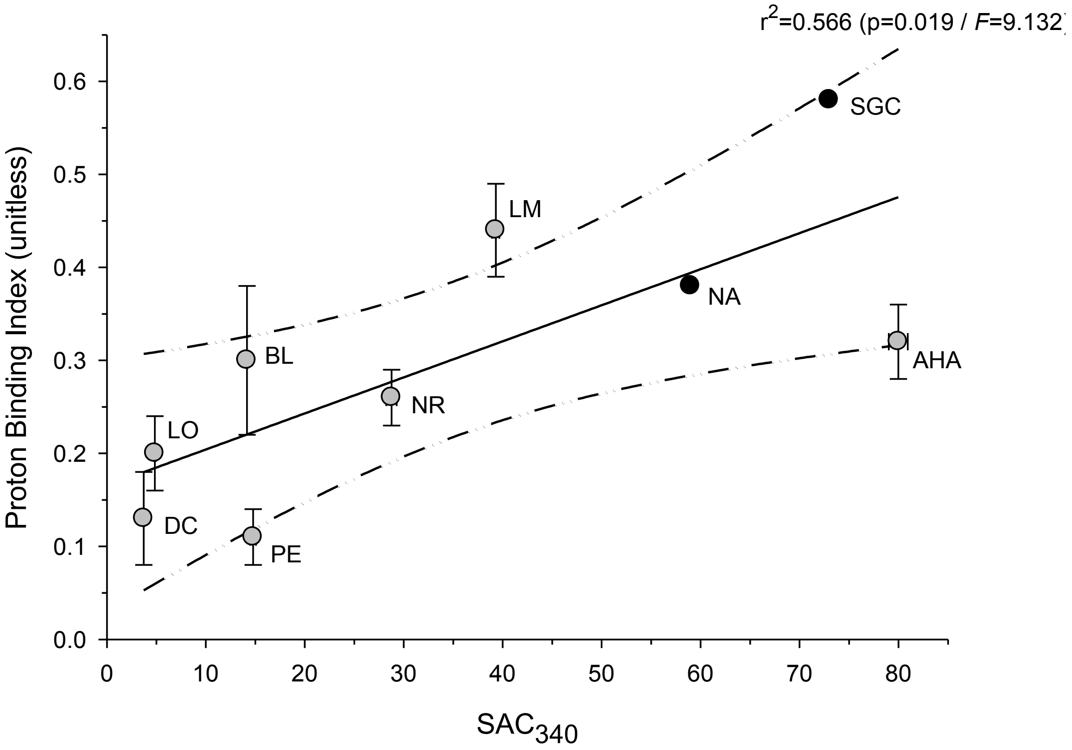

Figure S2. Proton Binding Index (PBI) versus SAC_{340} of both Rio Negro DOC samples and DOC from different sources previously analyzed by Al-Reasi et al. (2013)[8]. Note that dark colored DOC from Rio Negro displayed both high SAC_{340} and high PBI, which fits with the prediction of enhance ability to bind metals, and to affect gill physiology in aquatic animals. Dashed lines indicated 95% confidence intervals about the solid regression line. Nonlinear regression analysis was performed using *Sigma Plot* v 11.0. $r^2 = 0.566$; $p = 0.019$; $F = 9.132$
